# Pichinde virus induces microvascular endothelial cell permeability through the production of nitric oxide

**DOI:** 10.1186/1743-422X-6-162

**Published:** 2009-10-08

**Authors:** Rebecca L Brocato, Thomas G Voss

**Affiliations:** 1 Department of Microbiology and Immunology, Tulane University School of Medicine, New Orleans, LA, 70112, USA

## Abstract

This report is the first to demonstrate infection of human endothelial cells by Pichinde virus (PIC). PIC infection induces an upregulation of the inducible nitric oxide synthase gene; as well as an increase in detectable nitric oxide (NO). PIC induces an increase in permeability in endothelial cell monolayers which can be abrogated at all measured timepoints with the addition of a nitric oxide synthase inhibitor, indicating a role for NO in the alteration of endothelial barrier function. Because NO has shown antiviral activity against some viruses, viral titer was measured after addition of the NO synthase inhibitor and found to have no effect in altering virus load in infected EC. The NO synthase inhibition also has no effect on levels of activated caspases induced by PIC infection. Taken together, these data indicate NO production induced by Pichinde virus infection has a pathogenic effect on endothelial cell monolayer permeability.

## Introduction

Several members of the *Arenaviridae *family are the agents responsible for hemorrhagic fevers. These members include Junin virus, Machupo virus, and Lassa virus; the etiological agents of Argentine hemorrhagic fever (AHF), Bolivian hemorrhagic fever (BHF), and Lassa fever (LF), respectively [1]. Pichinde virus (PIC) belongs to the New World arenavirus complex along with Junin and Machupo [2]. However, unlike Junin and Machupo, PIC is not a human pathogen and therefore does not require high containment facilities to work with this virus. Due to this fact, other groups have used PIC as a model virus for arenavirus infection. Guinea pig infection with PIC has shown pathological similarities with LF, further supporting its use as a model for human Lassa fever [3].

The hallmark of infection by hemorrhagic fever viruses is the induction of vascular leak, or the breakdown of endothelial cell barrier function [4]. Endothelial cells are critical to vascular integrity by providing both structure and regulation of immune cells, solutes, and water across the barrier [5]. Vascular leak can be caused by direct viral effects that alter barrier integrity, the induction of apoptosis of the endothelium, or indirectly through the effects of soluble mediators such as pro-inflammatory cytokines created by the host immune response [4]. TNF-α and IFN-γ have been shown previously to induce vascular leak in a transendothelial resistance assay [6].

In general, arenaviruses are not highly cytopathic viruses *in vitro *or *in vivo *[7-9]. Therefore, it is believed that immune mediators play a significant role in endothelial cell barrier function. Previous studies of PIC have shown elevated levels of proinflammatory cytokines, such as TNF-α, during the course of infection of guinea pigs [10]. TNF-α has also been noted in Argentine hemorrhagic fever patients [11,12]. Other inflammatory mediators such as IL-8, IFN-γ, IL-12, IL-6, IP-10, and RANTES have been noted in the serum of LF patients [13].

Nitric oxide (NO) is a free radical with diverse physiological functions in humans. NO is a critical component of the innate immune response to various pathogens such as bacteria, parasites, and viruses including influenza A virus and coxsackie virus [14]. In addition to its role as in anti-microbial defense, NO has key roles in regulation of endothelial cell barrier function. Basal levels of NO are necessary for vasodilation, platelet aggregation, and the modulation of inflammatory cell adhesion to the endothelium [14-16]. The effects of NO on the cardiovascular system are dependent upon the amount of NO produced, the local environment, and redox state of NO. While low levels of NO are necessary for the integrity of the endothelium, excessive amounts of NO are pathogenic leading to compromised barrier function [17].

NO production has been noted in virulent Junin virus infection of endothelial cells *in vitro*. Serum samples from AHF patients confirm the increase in NO *in vivo*. By comparing these results to endothelial cells infected with non-virulent Junin virus, Gomez, et al, hypothesized that the increased production of NO was a contributing factor to the pathogenesis of AHF [18].

This study evaluated the potential of PIC to infect and induce permeability in human endothelial cell monolayers. The ability of PIC to induce the production of NO and TNF-α in response to viral infection; correlating with the induction of vascular leak was also determined. Inhibitors of vascular leak were evaluated for their ability to alter virus-induced leak. Finally, a caspase assay was used to determine if PIC-infected endothelial cells have activated caspases; and determine if vascular leak inhibitors alter the levels of these caspases.

## Materials and methods

### Cells and Virus

The immortalized human dermal microvascular endothelial cell line (HMEC-1) was provided by Edward Ades at the United States Centers for Disease Control and Prevention (CDC, Atlanta GA) [19]. Cells were maintained in Clonetics Endothelial Growth Medium (EGM-MV) supplemented with hydrocortisone, human endothelial growth factor, fetal bovine serum, vascular endothelial growth factor, human fibroblast growth factor-B, insulin-like growth factor, ascorbic acid, gentamicin and amphotericin-B. Pichinde (PIC) virus (designated CoAn 3739) was obtained from ATCC, propogated in Vero cells and titered by plaque assay using standard methods. Mouse immune ascites fluid (MIAF) to PIC was obtained from the University of Texas Medical Branch.

### Indirect Immunofluorescence

HMEC-1 cells were grown to confluence on collagen-coated glass chamber slides and infected with PIC at a multiplicity of infection of 1.0. After 72 hrs, cells were fixed with 10% paraformaldehyde in PBS at 4°C for 10 min, and washed with PBS. A 10 min incubation with NH_4_Cl was used to reduce background fluorescence. Cells were permeabilized with 0.01% TX-100 for 15 min and subsequently blocked with 8% heat-inactivated goat serum diluted in PBS. Primary antibody concentration used was 1:200. A goat anti-mouse Alexa-Fluor 488 (Molecular Probes) secondary antibody was used at a concentration of 1:400. Prolong gold anti-fade with DAPI was used as an overlay, then covered with a coverslip. Visualization was done using a Zeiss AxioPlan 2 fluorescent microscope.

### Transendothelial Resistance (TEER) Assay

Electrical resistance across a monolayer of HMEC-1 cells was measured using the Endohm chamber and volt-ohm meter (World Precision Instruments). Cells were grown on 6 mm collagen-coated polycarbonate membrane inserts (Corning) with 0.1 ml media in the upper chamber and 0.6 ml media in the lower chamber. This system contains two concentric electrodes, one in the bottom of the Endohm chamber and the other attached to the cap. Voltage is measured by the upper electrode relative to the bottom electrode. Therefore, resistance can be measured in a reproducible manner. Blank measurements were taken using a membrane insert with media and no cells. Resistance measurements were corrected for the area of the membrane insert and blank measurements using the following formula (R_exp_-R_b_)*0.33 cm^2^. Once cells had reached confluency, indicated by a constant resistance measurement, cells were infected with PIC at a multiplicity of infection (MOI) 0.1, 1 or 3. Resistance measurements were taken every 24 hours. Permeability inhibition assays were conducted according to the same protocol with the inhibitor added prior to virus infection.

### iNOS RT-PCR

RNA from PIC-infected HMEC-1s was isolated using Trizol (Invitrogen) according to the manufacturers' protocol. Real-time PCR was conducted using the iCycler (BioRad) with the iScript SYBR Green RT-PCR kit (BioRad). 1 μl of extracted RNA was added to 25 μl of master mix, 1 μl of reverse transcriptase, and 30 nM of each forward and reverse primer. Nuclease-free water was added to bring the total volume up to 50 μl.

The primer set used for the human inducible nitric oxide synthase (iNOS) gene was synthesized from sequences published in the literature [20-22]. The primer sequences for iNOS are: 5'-TCTTGGTCAAAGCTGTGCTC-3' (forward primer) and 5'-CATTGCCA-AACCTACTGGTC-3' (reverse primer). The PCR reaction protocol includes a cDNA synthesis step (50°C, 10 min) followed by reverse transcriptase inactivation (95°C, 5 min); 40 PCR cycles (95°C, 10 sec and 55°C, 30 sec) followed by melt curve analysis. Fold up- or down-regulation was calculated using the ΔΔC_t _method using C_t _values from the iNOS and GAPDH (Qiagen) primer assay.

### Quantitation of TNF-α and Nitric Oxide

HMEC-1 cells were grown to confluency and infected with PIC at an MOI of 1. Supernatants of HMEC-1 cell cultures were collected at 24, 48, 72, and 96 hrs post infection. The detection and quantitation of TNF-α was determined using an enzyme-linked immunosorbent assay kit (BD). ELISA assays were conducted according to the manufacturers' instructions. Detection and quantitation of nitric oxide was conducted on cell culture supernatants using Griess reagent (Invitrogen) (0.5% sulfanilamide, 0.05% N-(1-napthyl) ethylenediamine dihydrochloride in 2.5% H_3_PO_4_) in equal volumes. Absorbance was measured at 540 nm.

### Caspase Activation

Apoptosis was assessed by caspase-3/7 activation using the Apo-ONE Homogeneous Caspase-3/7 Assay kit (Promega). Briefly, confluent HMEC-1 cells were treated with an inhibitor and subsequently infected with PIC at an MOI of 1. Cells were incubated for 72 hrs. The cells were then lysed using a bifunctional cell lysis/activity buffer containing a profluorescent caspase-3/7 substrate. After incubation for 1.5 hours, fluorescence was measured at an excitation wavelength of 485 nm and an emission wavelength of 535 nm.

## Results

### Susceptibility of HMEC-1s to PIC infection

**Figure 1:**
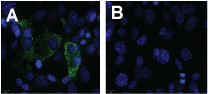
**Endothelial cell susceptibility to PIC infection**. HMEC-1 cells were seeded on collagen-coated chamber slides infected with PIC (A) at an MOI of 1, or mock infected (B), and incubated 72 hrs. Cells were indirectly immunostained with a PIC MIAF and an anti-mouse Ig-FITC. Magnification 63×.

Before determining the effects of PIC infection on endothelial cell barrier function, it was necessary to demonstrate HMEC-1 cell susceptibility to PIC infection *in vitro*. PIC antigen was detected by indirect immunofluorescence assay (IFA) using PIC specific antibody in the cytoplasm of infected cells (Fig. 1A). This is the first report to our knowledge of PIC infection of human endothelial cells.

### Effects of PIC infection on HMEC-1 permeability

**Figure 2:**
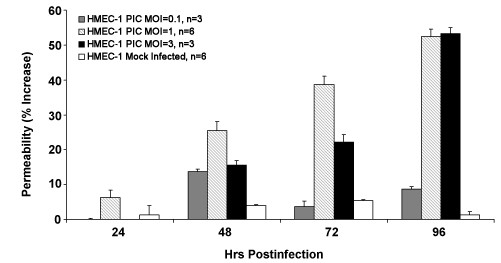
**Time dependence of PIC-induced permeability**. PIC induced permeability was quantified using the TEER assay. HMEC-1 cells were seeded onto collagen-coated membrane inserts and infected with PIC at MOI's of 0.1, 1, 3 or mock-infected. Each experimental condition was performed in triplicate. Results are expressed at % increase in permeability over basal permeability levels.

To investigate the effect of viral infection on endothelial cell permeability, HMEC-1s were grown to confluence on porous membrane inserts and subsequently infected with PIC. Permeability was measured using the TEER assay, which measures electrical resistance across a monolayer of endothelial cells. HMEC-1s infected with PIC at MOIs of 1 and 3 demonstrate a time dependent increase in PIC-induced permeability with a maximum reaching 60% 96 h post-infection. Infection of PIC at an MOI of 0.1 induced a maximum 15% increase in permeability 48 h post-infection (Fig. 2).

### NO and TNF-α Production by PIC-infected HMEC-1

**Figure 3:**
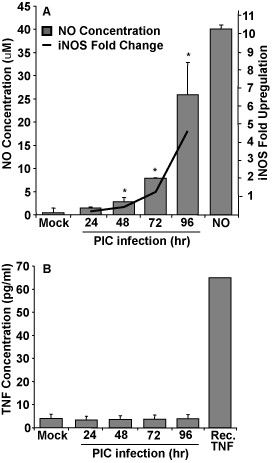
**Nitric oxide and TNF-α quantitation in PIC-infected HMEC-1 supernatants**. Supernatants collected from HMEC-1 cells infected at an MOI of 1 were assayed for nitrate/nitrite using Griess reagent (A, columns) as described above. Fold upregulation of the iNOS gene was determined by quantitative RT-PCR (A, line). TNF-α was assayed by ELISA. Each bar represents the mean of 3 independent experiments. * represents p < 0.05 when compared to mock-infected controls.

The protective role of low concentrations of NO in the vascular endothelium has been previously demonstrated [14,15,17]. To quantify the amount of NO produced by PIC-infected HMEC-1s, levels of nitrite/nitrate were determined by the addition of Griess reagent to cell culture supernatants. There is a statistically significant increase in NO by 48 h post-infection; increasing in supernatants collected at 72 and 96. These results are confirmed by RT-PCR results indicating an upregulation of the iNOS gene (Fig. 3A).

Because the production of TNF-α is a prominent feature in PIC-infected guinea pigs, the ability of PIC to induce TNF-α in HMEC-1 was assayed by ELISA. There was no significant increase in the amount of TNF-α produced by PIC-infected compared to mock-infected controls (Fig. 3B) supporting our hypothesis that other cell types, such as macrophages or dendritic cells, are responsible for the production of TNF-α in PIC infection.

### Effect of L-NAME on PIC-induced HMEC-1 permeability

**Figure 4:**
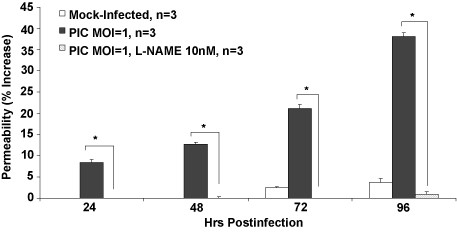
**Effect of L-NAME on PIC-induced permeability**. Prior to PIC infection, L-NAME was added to HMEC-1 cells at a concentration of 10 nM. Permeability was quantified as described above using the TEER assay. * represents p < 0.05 when compared to virus-infected HMEC-1.

The nitric oxide synthase inhibitor, N (G)-nitro-L-arginine methyl ester (L-NAME), was evaluated for its effects on PIC infected EC barrier function loss using the TEER assay. 10 nM of L-NAME added prior to PIC infection was sufficient to inhibit the increase in permeability induced by PIC to less than 2% (Fig. 4). This supports a significant role for NO in the increase in PIC-induced leak in HMEC-1.

### Effect of NO and L-NAME on PIC viral dynamics

**Figure 5:**
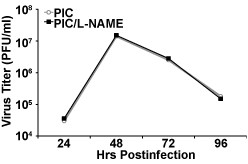
**Effect of L-NAME on PIC viral dynamics**. Supernatants from PIC-infected HMEC-1s treated with L-NAME were assayed for PIC by standard plaque assay. Each timepoint represents the mean of two experiments.

In order to determine the effect of NO production and inhibition on PIC replication in HMEC-1, supernatants from PIC-infected HMEC-1s that were treated with L-NAME were assayed for viral titer by plaque assay. There was no significant difference in the viral load of PIC in HMEC-1 indicating that NO production by infected EC did not have a protective role or limit PIC infection in HMEC-1 (Fig. 5).

### Effect of PIC on caspase activation

**Figure 6:**
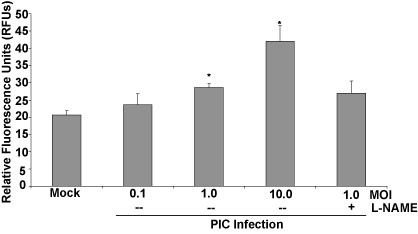
**Effect of PIC infection on caspase activation**. HMEC-1s were infected with PIC at MOIs of 0.1, 1, 10 (n = 3). In addition, 10 nM of L-NAME was added prior to PIC MOI = 1 infection (n = 5). Caspase activation was fluorescently detected 72 h post-infection. * represents p < 0.05 when compared with mock-infected controls. HMEC-1s treated with L-NAME and infected with PIC were tested for significance versus HMEC-1s infected with PIC MOI = 1; results were not considered significant.

Because the production of NO by PIC-infected HMEC-1s may create a cytotoxic environment, causing cells to undergo apoptosis ultimately leading to endothelial cell monolayer permeability changes, an examination of pathways associated with apoptosis was performed. Fluorescent detection of activated caspases indicates PIC induces an increase in activated caspases-3 and -7 at MOIs of 1 and 10 (Fig. 6). Addition of L-NAME at concentrations that block PIC-induced leak did not reduce the levels of activated caspases in HMEC-1. These results support other mechanisms of caspase activation, and not NO production in PIC-infected EC.

## Discussion

Lassa fever, the most prominent VHF of the *Arenaviridae *family, causes significant morbidity and mortality in West Africa. Lassa virus, along with Junin, Machupo, Guanarita, and Sabia, are considered Category A bioterrorism agents by the CDC. These viruses require BSL-4 containment that makes research on these viruses labor-intensive. PIC represents a non-pathogenic (for humans) model of arenavirus infection, which requires BSL-2 containment facilities. PIC virus adapted to strain 13 guinea pigs show pathological similarities with arenavirus-induced hemorrhagic disease, further supporting its utility as a model for evaluation of potential antiviral or other therapeutic targets for the treatment of virus-induced hemorrhagic disease.

Previously published studies demonstrate PIC-infected guinea pigs express elevated levels of TNF-α [3]. Evaluation of supernatant fluids from PIC-infected HMEC-1 by ELISA showed no increase in production of TNF-α compared to uninfected HMEC-1. These results indicate a non-TNF-α dependant mechanism of leak in PIC infected HMEC-1. Previous studies show PIC infection of murine macrophages leads to NF-κB activation, and the production of TNF-α and IL-6 [23]. Arenaviruses have been demonstrated to be macrophage tropic in vivo, we propose that macrophages as a primary source of TNF-α in PIC infection.

We have demonstrated PIC infection of human endothelial cells; and that PIC infection of HMEC-1s induces the production of NO. Analysis of the iNOS gene indicates an upregulation that correlates with NO levels produced. Elevated levels of NO have also been noted in Junin infection of endothelial cells, further supporting the utility of PIC infected HMEC-1 as a model for arenavirus-induced VHF. PIC induces an increase in permeability measured by the TEER assay. This increase in permeability can be abrogated with the addition of L-NAME, the NO synthase inhibitor. This shows that levels of NO produced by PIC-infected HMEC-1s are pathogenic and compromise endothelial cell barrier function.

NO has been shown to play a role in host defense against a variety of microbial pathogens: bacteria, parasites, and a variety of viruses [24]. Some of these viruses include influenza, coxsackievirus, rhinovirus, and vaccinia virus [25-28]. Studies conducted using the NO donor, SNAP, demonstrated that NO inhibits the synthesis of viral RNA in influenza virus infected cells, an early event in influenza replication. Comparing viral titers quantitated by plaque assay, it was determined that there was no significant change in viral titer when L-NAME was added to PIC-infected HMEC-1s. Studies conducted using lymphocytic choriomeningitis virus (LCMV), another arenavirus, show there no difference in viral kinetics, viral clearance, or the production of cytokines and chemokines in LCMV-infected iNOS knockout mice were compared with LCMV-infected wild type mice [29].

NO is a unique radical that demonstrates pro-apoptotic or anti-apoptotic effects [15]. A fluorometric assay was used to quantitate activated caspases that indicated an increase in PIC infected HMEC-1s. However, L-NAME was not able to significantly impact these levels. This demonstrates that the increase in NO is not responsible for the increase in activated caspases due to PIC infection.

In conclusion, these studies identify specific virus/cell interactions leading to vascular permeability in PIC infected HMEC-1. We demonstrate HMEC-1 cells are susceptible to PIC infection. PIC infection induces the production of NO. NO was determined to be an important factor in the loss of endothelial cell monolayer integrity. Inhibition of NO activity with L-NAME supported the role of NO in HMEC-1 leak. These studies will be critical as an *in vitro *model of VHF pathogenesis and to identify mechanisms of vascular leak and to identify potential inhibitors of VHF-induced leak in an effort to alleviate the severity of VHF.

## Competing interests

The authors declare that they have no competing interests.

## Authors' contributions

RLB carried out this study. RLB and TGV drafted the manuscript. All authors read and approved the final manuscript.
